# The Interferon Gamma-Related Long Noncoding RNA Signature Predicts Prognosis and Indicates Immune Microenvironment Infiltration in Colon Adenocarcinoma

**DOI:** 10.3389/fonc.2022.876660

**Published:** 2022-06-07

**Authors:** Cong Liu, Dingwei Liu, Fangfei Wang, Jun Xie, Yang Liu, Huan Wang, Jianfang Rong, Jinliang Xie, Jinyun Wang, Rong Zeng, Yong Xie

**Affiliations:** ^1^ Department of Gastroenterology, The First Affiliated Hospital of Nanchang University, Nanchang, China; ^2^ Gastroenterology Institute of Jiangxi Province, Nanchang, China; ^3^ Key Laboratory of Digestive Diseases of Jiangxi Province, Nanchang, China

**Keywords:** colon adenocarcinoma, lncRNA, IFN-γ, tumour immune microenvironment, prognosis

## Abstract

Colon adenocarcinoma (COAD) is one of the most common clinically malignant tumours of the digestive system, with high incidence and mortality and poor prognosis. Interferon-gamma (IFN-γ) and long noncoding RNAs (lncRNAs) have prognostic values and were closely associated with immune microenvironment in COAD. Thus, identifying IFN-γ-related lncRNAs may be valuable in predicting the survival of patients with COAD. In this study, we identified IFN-γ-related lncRNAs and divided COAD patients from the Cancer Genome Atlas (TCGA) database into training and validation sets. Pearson’s correlation analysis and least absolute shrinkage and selection operator (LASSO) Cox regression were performed to select IFN-γ-related lncRNA-associated prognoses. Thirteen lncRNAs (AC025165.8, AC091633.3, FENDRR, LINC00882, LINC01828, LINC01829, MYOSLID, RP11-154H23.4, RP11-20J15.3, RP11-324L17.1, RP11-342A23.2, RP11-805I24.3, SERTAD4-AS1) were identified to construct an IFN-γ-related lncRNA prognostic signature in TCGA training (n =213) and validation (n =213) cohorts. COAD patient risk scores were calculated and classified into high- and low-risk groups based on the median value of the risk scores in each dataset. We compared the overall survival (OS) of patients stratified by age, gender, and stage. The OS in the high-risk group was significantly shorter than that in the low-risk group. In addition, the clinical nomogram incorporating the prognostic signature and clinical features showed a high concordance index of 0.78 and accurately predicted 1-, 3-, and 5-year survival times among COAD patients in the high- and low-risk groups. Based on the risk model, the high- and low-risk groups exhibited distinct differences in the immune system by gene set enrichment analysis (GSEA) functional annotation, and differentially expressed genes (DEGs) between the high- and low-risk groups were subjected to Gene Ontology (GO) and Kyoto Encyclopedia of Genes and Genomes (KEGG) pathway enrichment analysis. We investigated the expression of multiple immune checkpoint genes in the high- and low-risk groups and plotted Kaplan-Meier survival curves, indicating that immune checkpoint genes, such as LAG3 and PD. L1, STING and TIM 3, were also expressed differently between the two risk groups. Subsequently, there were dramatic differences in mutated genes, SNV (single nucleotide variants) classes, variant types and variant allele frequencies between low- and high-risk patients with COAD. Patients stratified by risk scores had different sensitivities to common chemotherapeutic agents. Finally, we used quantitative real-time polymerase chain reaction (qRT-PCR) assays to demonstrate that three lncRNAs were significantly differentially expressed in COAD tissues and adjacent normal tissues. Considered together, a thirteen-lncRNA prognostic signature has great potential to be a prognostic biomarker and could play an essential role in the immune microenvironment of COAD.

## Introduction

Colon adenocarcinoma (COAD) is a prevalent malignant tumour of the digestive tract that causes almost 900,000 deaths each year and has become the world’s fourth most deadly cancer worldwide (1). Significant advances have been achieved in comprehensive treatments for COAD, including diagnosis, surgery, chemotherapy, radiotherapy, targeted therapy, adjuvant therapy and molecular-targeted drug therapy (2). Nevertheless, the 5-year survival rate of COAD patients remains unsatisfactory due to chemoresistance and high rates of distant metastasis. Hence, it is of great priority to identify accurate and efficient early diagnosis and prognosis biomarkers for patients with COAD.

Interferon-gamma (IFN-γ) is a pluripotent cytokine produced by multiple constituents of immune cell subsets, including natural killer (NK) cells, natural killer T cells (NKT) cells, gamma delta T cells, CD4^+^ T helper cells and CD8^+^ cytotoxic T cells (3). Numerous properties of IFN-γ have been elucidated, including antitumour, antiviral, antiproliferative, and immunomodulatory effects (4). Over the past few years, a growing body of research has demonstrated that IFN-γ participates in the initiation and progression of COAD (5). Despite these findings, no relevant studies have been conducted to comprehensively analyse and screen for IFN-γ-related lncRNAs as risk signatures for COAD prognosis.

Long noncoding RNAs (lncRNAs) are a class of gene transcription RNAs consisting of more than 200 nucleotides without protein-coding potential (6). Accumulating evidence has suggested that lncRNAs are widely involved in diverse biological functions, such as the translation of cytoplasmic mRNAs, protection of genome integrity, immune response and regulation of heterochromatin formation (7–9). Currently, certain lncRNAs have served as tumour biomarkers, including PCA3, HULC, and MALAT1 (10). Consequently, it would be of great significance to identify novel prognostic biomarkers and develop therapeutic targets in COAD.

In the present study, we downloaded and integrated the gene expression data and the clinical information of patients with COAD from TCGA database, IFN-γ-related lncRNA prognostic signature was obtained by integrated bioinformatics and statistical analysis. Then, 13 lncRNAs with strong correlation were filtrated and used to construct the IFN-γ-related lncRNA prognostic signature. Afterwards, COAD patients risk scores were calculated and classified into high- and low-risk groups based on the median risk score in each dataset. Moreover, we combined predictable clinical features with risk score to construct an efficient nomogram to predict the survival rate of COAD patients. Then, we preliminarily revealed the differences in the immune microenvironment in high- and low-risk groups by CIBERSORT algorithm and the expression levels of immune checkpoints genes. Furthermore, we performed sensitivity analyses of common chemotherapeutic agents for COAD patients classified by risk scores. To the end, we further verified that three lncRNAs’ expressions were significantly different between COAD tissues and adjacent normal tissues by qRT-PCR assays. Collectively, we aimed to take advantage of the lncRNA expression profiles to explore novel prognostic predictors and their correlations in the COAD immune microenvironment, which may promote better personalized treatment strategies and shed light on underlying mechanisms.

## Materials and Methods

### Data Extraction and Processing

RNA sequence transcriptome data, lncRNA annotation files and clinical information of COAD patients were obtained from the TCGA (https://cancergenome.nih.gov) database. The details can be found in **Supplementary Tables 1–3**. The gene set ‘HALLMARK_INTERFERON_GAMMA_RESPONSE’, which includes 200 genes in response to interferon gamma, was obtained from the molecular signature database of Gene Set Enrichment Analysis (GSEA) (https://www.gseamsigdb.org/). The symbols of these 200 genes were shown in **Supplementary Table 4**. The average RNA expression value was used when duplicate data were found.

### Identification of IFN-γ-Related lncRNAs

Significant differential expression of lncRNAs and interferon gamma was identified by the “limma” package in R software with |log_2_FC|≥1 and FDR<0.05. Then, Pearson’s correlation analysis was performed to determine the correlations between lncRNAs and interferon gamma. lncRNAs with a correlation coefficient |R2|>0.4 and P<0.05 were considered IFN-γ-related lncRNAs.

### Establishment and Validation of an IFN-γ-Related lncRNA Prognostic Signature

The entire TCGA dataset was randomly assigned into training and validation cohorts. Patients with fewer than 30 days of overall survival (OS) were excluded. A total of 426 COAD patients were randomly divided into a training cohort (n=213) and a validation cohort (n=213). The training cohort was used to build an IFN-γ-related lncRNA model, and the validation cohort was employed to validate the model. Thereafter, least absolute shrinkage and selection operator (LASSO) Cox regression analysis of IFN-γ-related lncRNAs was conducted using the R package “glmnet”. We established an IFN-γ-related lncRNA prognostic signature for COAD patients composed of 13 IFN-γ-related lncRNAs. The risk score of each patient was calculated based on the following computational formula: Risk score = coeff (lncRNA1) × expr (lncRNA1) +coeff (lncRNA2) ×expr (lncRNA2) + …… + coeff (lncRNAn) × expr (lncRNAn). The coeff represents the coefficients, coeff (lncRNAn) indicates the coefficient of lncRNAs correlated with survival, and expr (lncRNAn) is defined as the expression of lncRNAs. According to the median risk score, COAD patients in the TCGA dataset were divided into high- and low-risk groups.

### Nomogram Construction

The predictive ability of the nomogram was constructed by integrating traditional clinical features, such as age, stage and risk score. The nomogram was used to predict the 1-, 3-, and 5‐year OS of COAD patients. The calibration curves of the nomogram were generated to assess the predictive accuracy of the prognostic signature.

### Gene Set Enrichment Analysis

The DEGs between the high- and low-risk groups were subjected to Gene Ontology (GO) and Kyoto Encyclopedia of Genes and Genomes (KEGG) pathway enrichment analysis. Additionally, we performed gene set enrichment analysis (GSEA) to determine different functional phenotypes between high- and low-risk group patients.

### Immune Cell Infiltration Assessment

To explore the relationships with immune cell infiltration, we used the CIBERSORT algorithm to calculate the infiltration expression of 22 immune cells in COAD cohorts. The enrichment proportion calculated by CIBERSORT represented the relative abundance of each immune cell in COAD samples.

### Chemotherapy Drug Susceptibility Analysis

We assessed the sensitivity of COAD patients to common chemotherapeutic agents through the Genomics of Drug Sensitivity in Cancer (GDSC; https://www.cancerrxgene.org/) database. To investigate the clinical performance of chemotherapeutic drugs, we determined the half maximal inhibitory concentration (IC50) of common chemotherapeutic agents using the R package “pRRophetic”.

### Samples and Quantitative Real-Time Polymerase Chain Reaction

We totally collected 11 pairs of COAD tissues and adjacent normal tissues from patients who underwent surgical procedures in The First Affiliated Hospital of Nanchang University. Harvested tissues were immediately frozen in liquid nitrogen and then stored at -80°C before RNA extraction. This research was approved by The First Affiliated Hospital of Nanchang University Ethics Committee on Medical Research. The sample acquisition and usage were carried out in accordance with the approved guidelines. Written informed consent was obtained for each patient. To assess the expression levels of lncRNAs in COAD patients, we used Trizol reagent (Invitrogen, Carlsbad, CA, United States) to extract total RNA according to the instructions of manufacturer. cDNA synthesis was performed by using reverse transcription kit (TIANGEN BIOTECH BEIJING CO., Ltd). The qRT-PCR analysis was conducted on Applied Biosystems QuantStudio 5. The expression level of lncRNAs were calculated using the 2^-△△Ct^ method and the related GAPDH mRNA expression was used as an endogenous control. The following primer sequences (Guangzhou Ribobio Co., Ltd): LINCAC025165.8 forward 5’-TGATTTGCTCAAAGAGGAAGACG-3’ and reverse 5’-TTTCTGGGTCACCGAGCCT-3’; LINC RP11-324L17.1 forward 5’-CGCTTCCAAGAGTGGCAATC-3’, and reverse 5’-CCTGTTTCCAAATGAGTCTGTCC-3’; SERTAD4-AS1 forward 5’- GGAAGGAACAAGATCAAGGATGA-3’, and reverse 5’- GCATGTCAGTCACCCAAGTTTTA-3’.

### Statistical Analysis

R software (version 4.0.5, https://www.r-project.org/) was used for all of the statistical analyses and plotting. The independent prognostic factors in COAD were identified using univariate and multivariate Cox regression analyses. The correlation between risk score and clinicopathological characteristics were compared by the Wilcoxon test. p<0.05 was considered statistically significant.

## Results

### Identification of IFN-γ-Related lncRNAs With Prognostic Value in COAD

**Figure 1:**
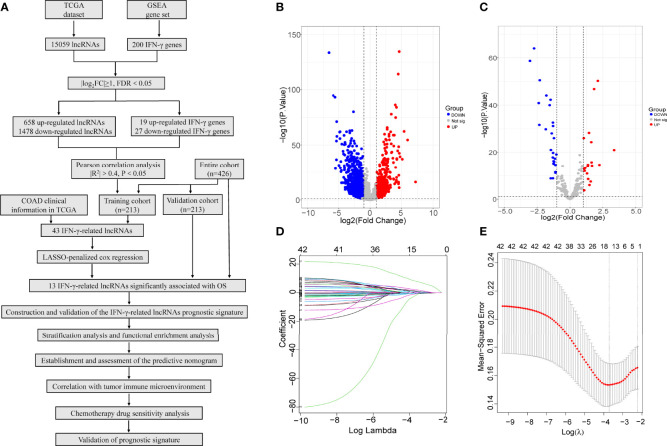
Identification of IFN-γ-related lncRNAs in COAD. **(A)** Flow chart of this study. **(B, C)** Among tumour samples and adjacent normal samples, a volcano diagram of differentially expressed lncRNAs **(B)** and IFN-γ related genes **(C)**. The vertical axis represents -log10 (P value), the horizontal axis represents differential expression multiple log2 (Fold Change), the blue colour indicates downregulated genes, and the red colour indicates upregulated genes. **(D)** The Least absolute shrinkage and selection operator (LASSO) coefficient profile of 13 OS-related lncRNAs and perpendicular imaginary line were drawn at the value chosen by 10-fold cross-validation. **(E)** The optimal tuning parameter (log λ) of prognostic IFN-γ-related lncRNAs were selected to cross-validation the error curve. The vertical axis is the mean-squared error, and the horizontal axis is log λ. The dotted vertical lines were plotted at the optimal value in accordance with the minimal criterion and 1-se criterion.

The flow chart of this study is displayed in **Figure 1A**. First, a total of 15,059 lncRNAs were abstracted from the TCGA database. We chose the gene set ‘HALLMARK_INTERFERON_GAMMA_RESPONSE’, which includes 200 genes in response to interferon gamma, from the molecular signature database of Gene Set Enrichment Analysis (GSEA) (https://www.gseamsigdb.org/). Among tumour samples and adjacent normal samples, a volcano diagram showed 658 upregulated and 1478 downregulated lncRNAs, 19 upregulated and 27 downregulated IFN-γ, identified by the “limma” package in R software (**Figures 1B, C**). Forty-three IFN-γ-related lncRNAs related to the survival of COAD patients were identified by Pearson’s correlation analysis *via* the criterion with |R^2^|>0.4 and P<0.05. Under LASSO-penalized Cox regression, 13 prognostic IFN-γ-related lncRNAs were further screened in the training group based on 1,000 times tenfold cross-validation (**Figures 1D, E**). Accordingly, the aforementioned study identified 13 IFN-γ-related lncRNAs with significant prognostic value for COAD.

### The Expression of 13 IFN-γ-Related lncRNAs With Prognostic Value

**Figure 2:**
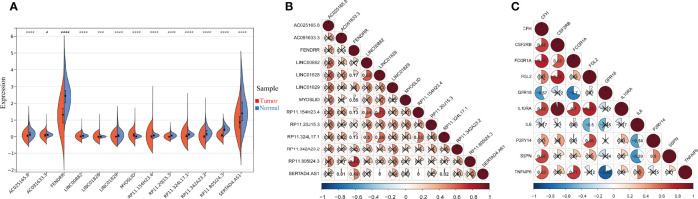
The expression and correlation among IFN-γ-related lncRNAs. **(A)** Violin plot of the expression levels of 13 IFN-γ-related lncRNAs between tumour and normal samples. (*p < 0.05; ***p < 0.001; ****p < 0.0001). **(B)** The correlation of 13 differently expressed IFN-γ-related lncRNAs from the TCGA COAD cohort. **(C)** The correlation among 10 differently expressed IFN-γ related genes.

In the preceding analysis, we identified 13 IFN-γ-related lncRNAs (AC025165.8, AC091633.3, FENDRR, LINC00882, LINC01828, LINC01829, MYOSLID, RP11.154H23.4, RP11.20J15.3, RP11.324L17.1, RP11.342A23.2, RP11.805I24.3, SERTAD4.AS1) and 10 IFN-γ related genes (CFH, CSF2RB, FCGR1A, FGL2, GPR18, IL10RA, IL6, P2RY14, SSPN, TNFAIP6). The expression of the 13 IFN-γ-related lncRNAs in the entire set was shown in **Figure 2A**, all lncRNAs’ expression in tumour samples and adjacent normal samples were statistically significant. The correlation analysis of the expression of the 13 IFN-γ-related lncRNAs is shown in **Figure 2B**. Clearly, we observed that RP11.805I24.3 had a strong correlation with FENDRR. Furthermore, the correlation analysis of the 10 IFN-γ related genes was also illustrated in **Figure 2C**, it is evidently observed that IL10RA was most significantly associated with CSFC2RB.

### Construction and Validation of the IFN-γ-Related lncRNA Prognostic Signature in COAD

**Figure 3:**
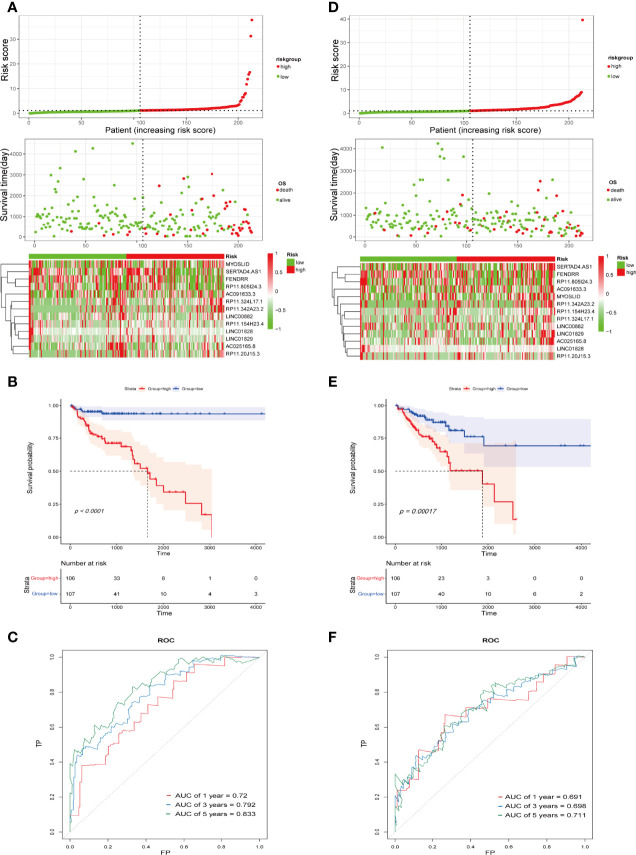
Construction and validation of the IFN-γ-related lncRNA prognostic signature in the training cohort and the validation cohort. **(A, D)** Correlation between the prognostic signature and the OS of patients in the training cohort **(A)** and validation cohort **(D)**. The distribution of risk score (upper), survival status (middle), and heatmap of selected IFN-γ-related lncRNAs (below). **(B, E)** Kaplan-Meier survival curves of the OS for COAD patients between the high- and low-risk groups in the training cohort **(B)** and validation cohort **(E)**. **(C, F)** ROC curves of the IFN-γ-related lncRNA prognostic signature for predicting 1-, 3-, and 5-year survival in the training cohort **(C)** and validation cohort **(F)**.

An IFN-γ-related lncRNA prognostic signature was constructed, composed of the 13 IFN-γ-related lncRNAs in the training cohort. COAD patients were separated into high- and low-risk groups based on the median risk score. The distribution of risk scores in the two different risk groups is shown in **Figure 3A**. The survival status and survival time of COAD patients between the low-risk and high-risk groups are displayed in **Figure 3A**. The relative expression profiles of the 13 IFN-γ-related lncRNAs for each patient are exhibited in **Figure 3A**. Most conspicuously, the Kaplan-Meier survival curves showed that the low-risk group displayed better overall survival (OS) than the high-risk group (**Figure 3B**). As illustrated in **Figure 3C**, the areas under the receiver operating characteristic (ROC) curve (AUC) for 1-, 3-, and 5-year survival were 0.72, 0.792, and 0.833, respectively, in the training cohort, demonstrating that the signature has great potential as a prognostic indicator for COAD patients. To further validate the accuracy of the signature, we performed similar analyses in the validation cohort. The risk scores of each patient in the validation cohort were calculated by the uniform formula. Risk score distribution, the survival status and survival time of patients, and IFN-γ-related lncRNA expression profiles are shown in **Figure 3D**. Kaplan-Meier survival analyses indicated that the high-risk group displayed worse OS than the low-risk group (**Figure 3E**). The AUCs of 1-, 3-, and 5-year survival were 0.691, 0.698, and 0.711, respectively (**Figure 3F**).

To better assess the prognostic capacity of the IFN-γ-related lncRNA prognostic signature, stratification analysis was conducted to determine whether it maintained its ability to predict OS among various subgroups.

**Figure 4:**
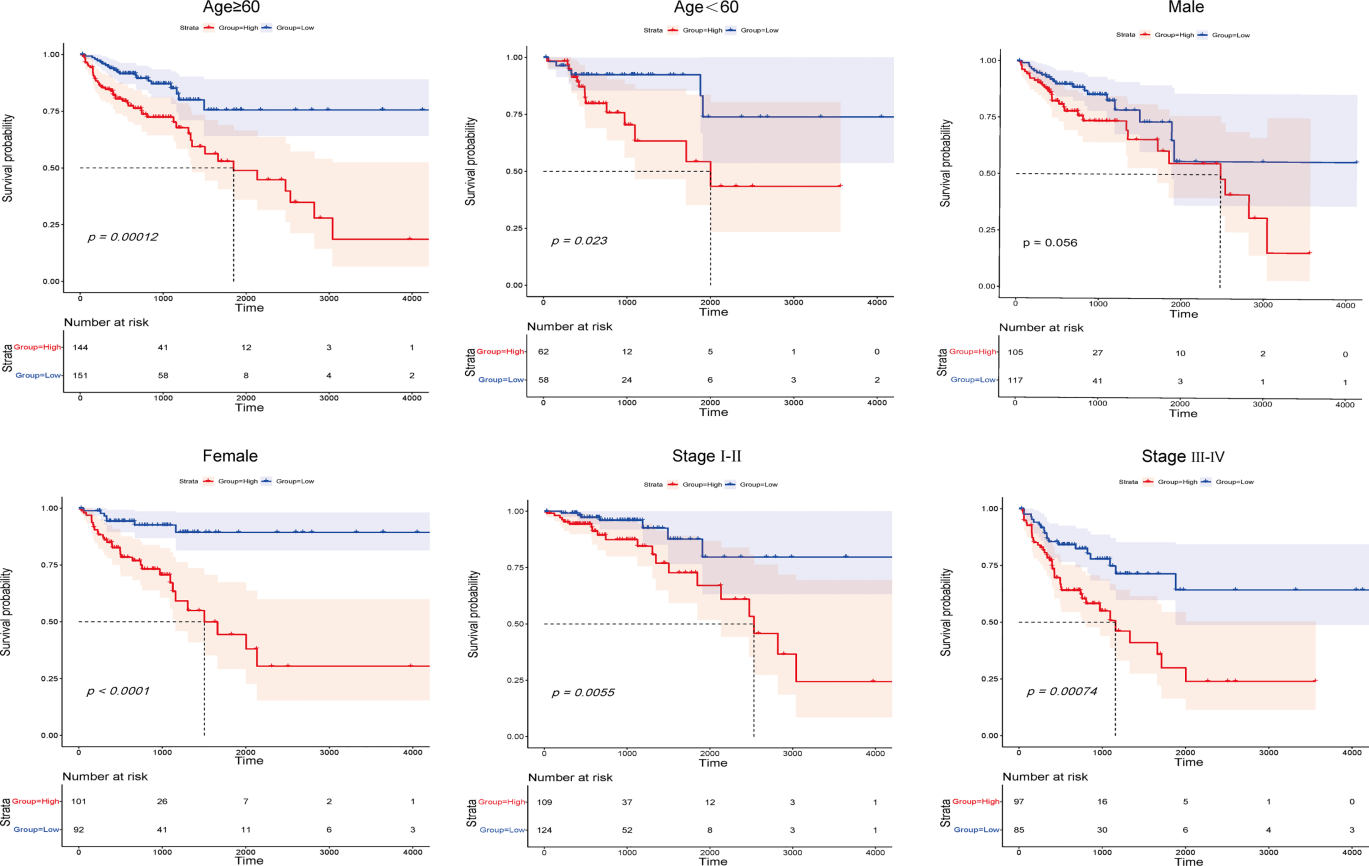
Kaplan-Meier survival curves of COAD patients stratified by age, gender, and tumour grade in the high- and low-risk groups.

On the basis of subgroups categorized by age, gender, and tumour stage, the OS of the low-risk group continued to be better than that of the high-risk group (**Figure 4**). Therefore, the studies described above identified 13 IFN-γ-related lncRNAs with prognostic value for COAD.

### Establishment and Assessment of the IFN-γ-Related lncRNA Prognostic Signature Based on a Nomogram Model

**Figure 5:**
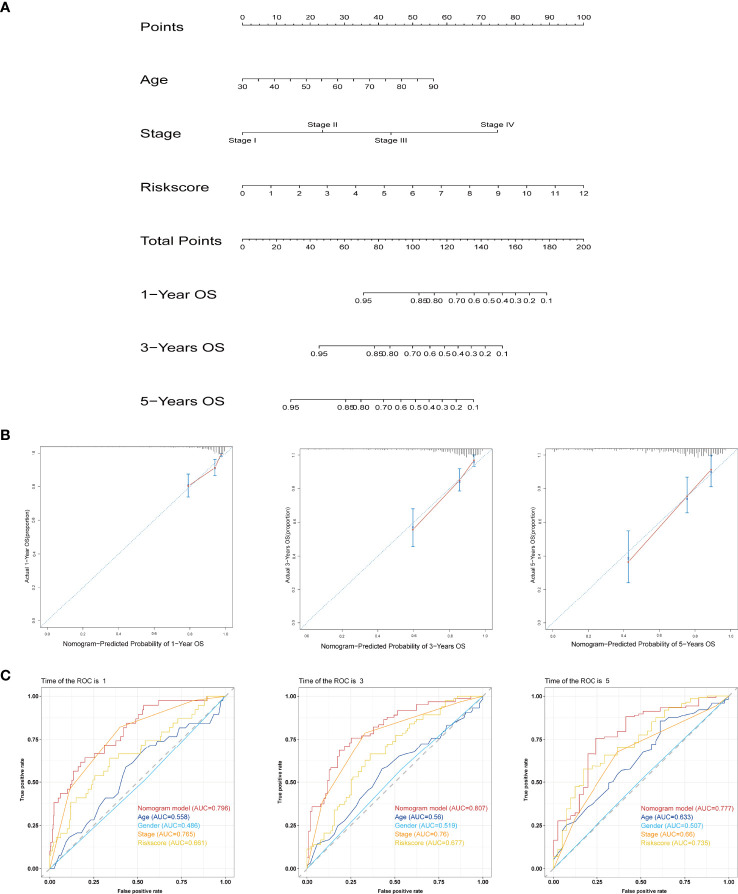
Establishment and assessment of the prognostic nomogram based on the IFN-γ-related lncRNA signature and clinicopathological features. **(A)** Nomogram for predicting the 1-, 3-, and 5-year prognosis of COAD patients. **(B)** Calibration curves of the nomogram for predicting the probability of OS at 1, 3, and 5 years. The X-axis represents nomogram predicted survival and the Y-axis represents the actual survival. **(C)** ROC curves of the nomogram for predicting OS at 1, 3, and 5 years.

We analysed the associations between clinicopathological parameters of COAD patients from TAGA database and the risk scores from the IFN-γ-related lncRNA prognostic signature. The heatmap demonstrated that RP11.342A23.2, AC091633.3, MYOSLID, RP11.20J15.3, RP11.324L17.1, and AC025165.8 expression increased with increasing risk score, whereas the expression of LINC01829 and LINC01828 decreased with increasing risk score. Their expression levels were also associated with the clinicopathological characteristics of COAD, such as survival status, stage, gender, and age (**Supplementary Figure 1A**). Then, we analyse the relationships between the risk scores and age, gender, tumour stage. As shown in **Supplementary Figure 1B**, our result shown that the correlation of age and risk score was not statistically significant (p = 0.16). Meanwhile, **Supplementary Figure 1C** illustrated that the correlation of gender and risk score was also not statistically significant (p = 0.78). As shown in **Supplementary Figure 1D**, the risk scores are statistically significant different between of stage I and stage II (p < 0.05), the risk score of stage I and stage III was statistically significant different (p < 0.05), the risk score of stage I and stage IV was statistically significant different (p < 0.01). Collectively, the risk score of different stage was different, the correlation of stage and risk score was statistically significant. These results demonstrate some demographic and clinical characteristics that are sensitive to the IFN-γ-related lncRNA prognostic signature and further confirm the clinicopathological application value of the model. Subsequently, univariate and multivariate Cox regression analyses were employed to reveal whether the IFN-γ-related lncRNA prognostic signature was an independent prognostic factor for COAD patients. Univariate Cox regression analysis illustrated that the IFN-γ-related lncRNA prognostic signature was remarkably associated with OS [hazard ratio (HR): 1.360, 95% CI: 1.250–1.481, p < 0.001; **Supplementary Figure 2A**]. Additionally, multivariate Cox regression analysis further suggested that the IFN-γ-related lncRNA prognostic signature could independently predict the prognosis of COAD patients (HR: 1.342, 95% CI: 1.223–1.472, p < 0.001; **Supplementary Figure 2B**). A nomogram integrating clinicopathological features and IFN-γ-related lncRNA prognostic signatures was constructed to evaluate the 1-, 3-, and 5-year OS (**Figure 5A**). The calibration curve indicated that the actual observation vs. prediction rates of 1-, 3- and 5-year OS demonstrated a good consensus (**Figure 5B**). Furthermore, The AUC values of nomogram for 1-, 3-, and 5-year OS were 0.796, 0.807, and 0.777, respectively, demonstrating that it had excellent predictive capability for prognosis (**Figure 5C**).

### Functional Analysis of the Signature

**Figure 6:**
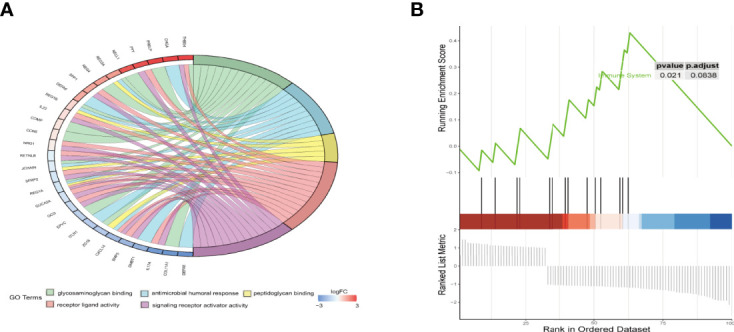
Functional enrichment analysis of DEGs between the high- and low-risk groups. **(A)** The chord plot of GO enrichment analysis. **(B)** Gene set enrichment analysis (GSEA) of hallmark gene sets regarding DEGs between high- and low-risk groups.

First, GO analysis and KEGG pathway enrichment analysis of DEGs between high- and low-risk groups were conducted. As shown in **Figure 6A**, the top five significant GO terms of DEGs were “glycosaminoglycan binding,” “antimicrobial humoral response,” “peptidoglycan binding,” “receptor ligand activity,” and “signalling receptor activator activity”. The KEGG pathway enrichment results are displayed in **Supplementary Figure 3**, we observed that these DEGs was mainly enriched in ECM-receptor interaction. GSEA indicated significant differences in the immune system between the high- and low-risk groups (**Figure 6B**). The above analyses unambiguously revealed that the prognostic signature could accurately distinguish between high- and low-risk groups.

### Interrelationship Between IFN-γ-Related lncRNA Prognostic Signature and Immune Cell Infiltration

**Figure 7:**
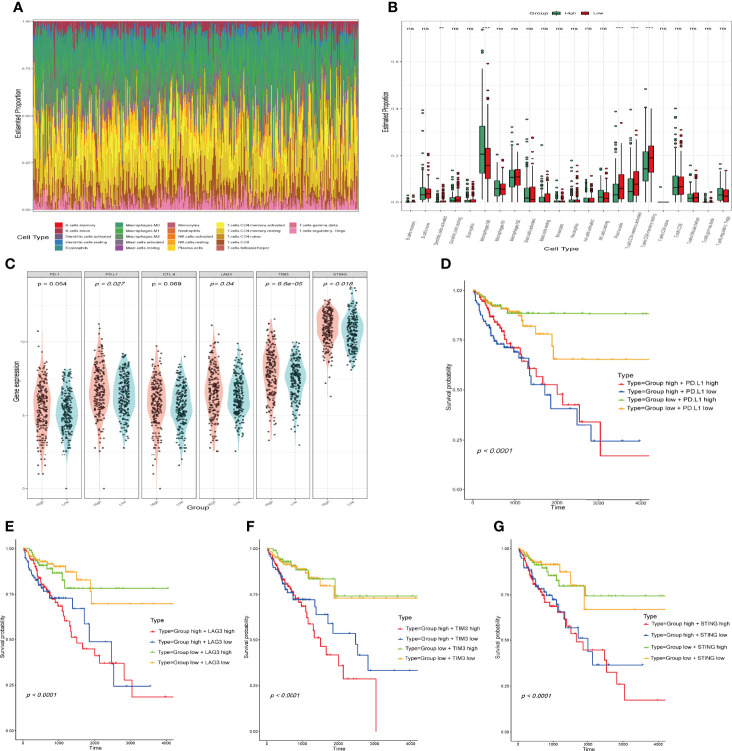
Comparison of immune infiltration status and immune checkpoint gene expression between the high- and the low-risk groups. **(A)** Relative proportion of tumour‐infiltrating immune cells in all patients. **(B)** Differences in the infiltrating levels of 22 tumour-infiltrating immune cell types between the high- and low-risk groups by using the CIBERSORT algorithm. (**p < 0.01; ***p < 0.001; ns, not significant). **(C)** The expression of immune checkpoint genes in the high- and low-risk groups. The horizontal axis is the high and low risk group, the longitudinal axis is the expression of immune checkpoint genes. **(D–G)** Kaplan-Meier survival curves of overall survival among four patient groups stratified by the IFN-γ-related lncRNAs signature and PD. L1 **(D)**, LAG3 **(E)**, TIM3 **(F)** and STING **(G)**.

We analysed the correlation between the IFN-γ-related lncRNA prognostic signature and 22 tumour-infiltrating immune cells using the CIBERSORT algorithm. The immune landscape of predominant immune cell types in COAD is presented in **Figure 7A**. As shown in **Figure 7B**, COAD patients were separated into high- and low-risk groups based on the median risk score. The results showed that B cells, macrophages, resting CD4^+^ T cells, activated CD4^+^ T cells and CD8^+^ T cells occupied a substantial proportion of immune cell infiltration. Regarding the number of macrophages, M0 was significantly higher in the high-risk group than in the low-risk group. Conversely, the numbers of plasma cells, activated memory CD4^+^ T cells and resting memory CD4^+^ T cells were significantly lower in the high-risk group than in the low-risk group. Given the importance of controlling the expression of immune checkpoint genes for the treatment of COAD, we further assessed the differential expression of immune checkpoint genes in both groups, our results revealed that patients in the low-risk group have a lower expression of immune checkpoint genes than patients in the high-risk group (**Figure 7C**). In addition, we analysed survival distribution of four patient groups stratified by the high/low immune checkpoint genes expression and the high/low-risk score. Kaplan-Meier survival curves indicated that patients in the high-risk group with high PD. L1 have worse survival rates than patients in the low-risk group with high PD. L1, and patients in the high-risk group with low PD. L1 have worse survival rates than patients in the low-risk group with low PD. L1 (**Figure 7D**). The similar results in LAG3, TIM3 and STING stratified groups were also observed (**Figures 7E–G**). In total, our analysis demonstrated that the IFN-γ-related lncRNA prognostic signature might be implicated in the tumour immune microenvironment.

### Differentiation of Mutated Genes Between High- and Low-Risk Groups

**Figure 8:**
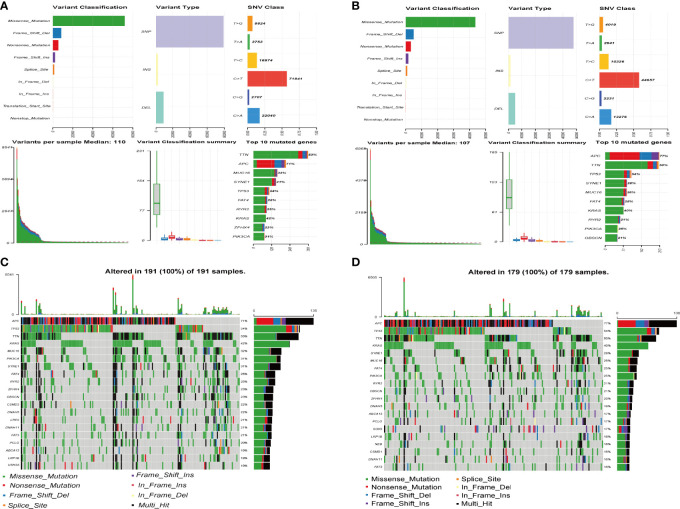
Mutation information of mutated genes between high- and low-risk groups. **(A, B)** The distribution of different types of mutations between the high-risk **(A)** and low-risk groups **(B)**. The upper represents variant classification, variant type, SNV (single nucleotide variants) class of mutated genes, the blow represents variants per sample, variant classification, top 10 mutated genes. **(C, D)** Waterfall plot of the top 20 mutated genes in the high-risk **(C)** and low-risk groups **(D)**. The gene mutation patterns for each sample are displayed in the middle panel. The upper bar plot depicts the total mutation burden of each sample. The mutation frequencies for each gene are shown in the right panel.

As presented in **Figures 8A, B**, we analysed and summarized the mutation information of mutated genes based on variant type, SNV class, variants per sample and variant classification. The mutational landscape of the top 20 genes with the highest mutation frequencies in the high- and low-risk groups was revealed in the waterfall plot. In the high-risk group, we found that APC exhibited the highest mutation frequency, accounting for 71%, followed by TP53, TTN, KRAS, MUC16, PIK3CA, and SYNE1 (**Figure 8C**). In the low-risk group, the expression levels of 20 mutated genes, including APC (77%), TP53 (54%), and TTN (50%), were higher than those of others altered in 179 (100%) of 179 samples (**Figure 8D**).

### Chemotherapy Drug Sensitivity Analysis

**Figure 9:**
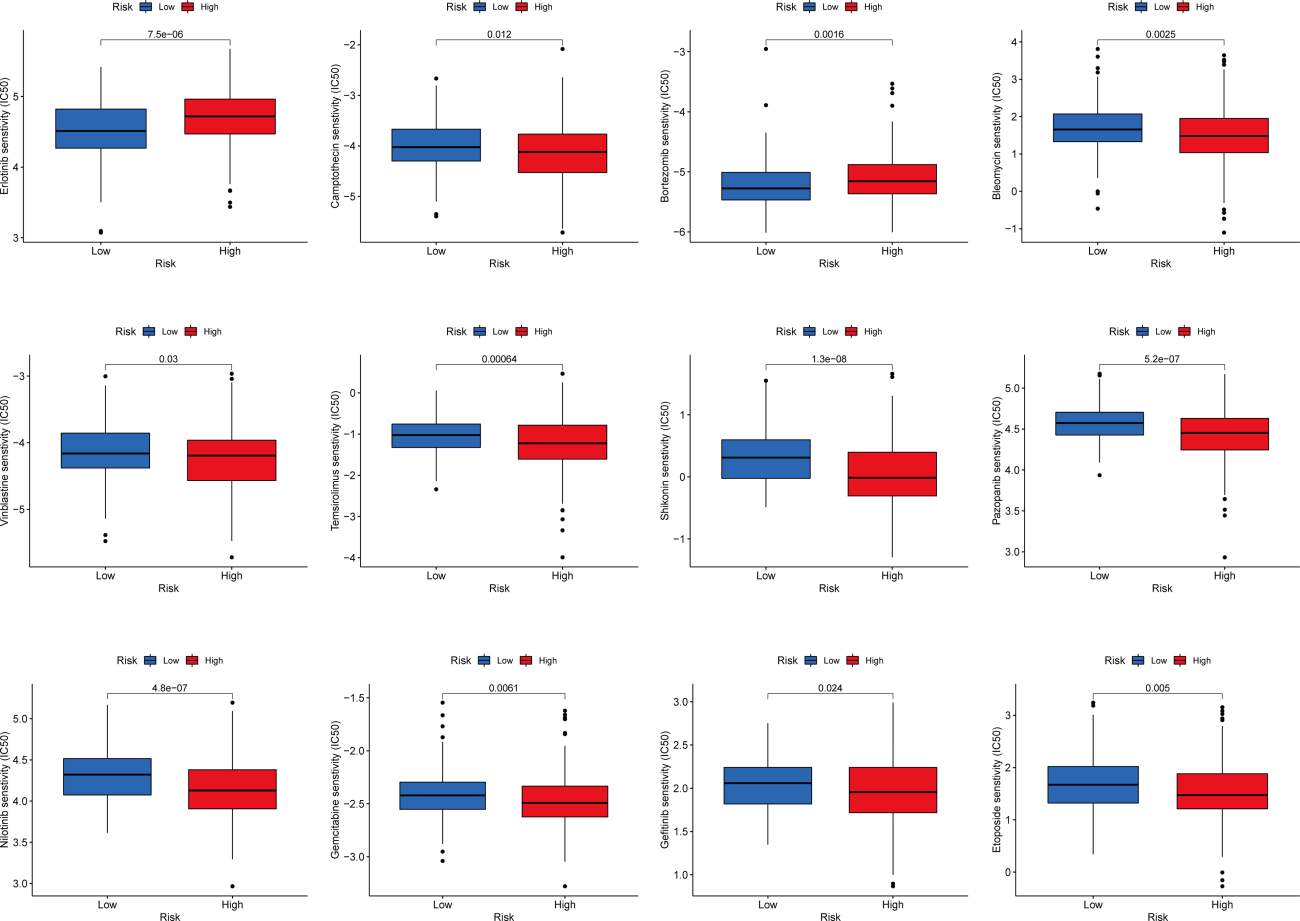
Relationships between the IFN-γ-related lncRNAs signature and chemotherapy response.

We explored the sensitivity of COAD patients stratified by risk scores to the common chemotherapeutic agents. The IC50 values of common chemotherapeutic drugs were calculated by the pRRophetic algorithm. We found that low-risk patients had higher estimated IC50 values for camptothecin, bleomycin, vinblastine, temsirolimus, shikonin, pazopanib, etoposide, gefitinib, gemcitabine, and nilotinib, while high-risk patients had higher estimated IC50 values for erlotinib and bortezomib. In brief, these results indicated that the IFN-γ-related lncRNA prognostic signature was related to drug sensitivity. (**Figure 9**).

### Validation of the Expression Levels of Three IFN-γ-Related lncRNAs in COAD Samples

**Figure 10:**
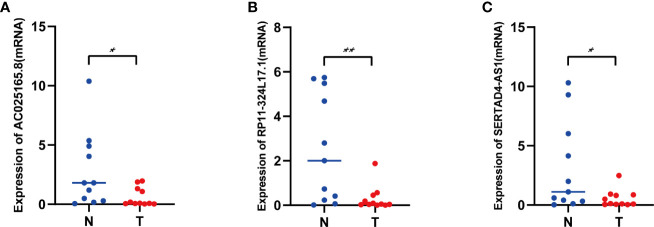
The expression levels of three lncRNAs in COAD tissues and adjacent normal tissues. **(A)** The expression levels of AC025165.8 in carcinoma tissues and adjacent tissues. **(B)** The expression levels of RP11-324L17.1 in carcinoma tissues and adjacent tissues. **(C)** The expression levels of SERTAD4-AS1 in carcinoma tissues and adjacent tissues. (*p < 0.05; **p < 0.01).

To evaluate the lncRNAs that are important for construction of the prognostic signature, we detected three IFN-γ-related prognostic lncRNAs expression levels in our collected 11 COAD tissues and adjacent normal tissues by using qRT-PCR assays. As shown in **Figure 10A**, AC025165.8 is highly expressed in adjacent normal tissues compared with COAD tissues. The expression levels of RP11-324L17.1, SERTAD4-AS1 showed similar results (**Figures 10B, C**). Besides, FENDRR was significantly upregulated in adjacent normal tissues compared with COAD tissues (11, 12).

## Discussion

COAD is one of the leading causes of cancer-associated death worldwide due to its late diagnosis, high recurrence rate and poor prognosis (13, 14). Although aggressive multimodal therapy (surgery, chemotherapy, immunotherapy, and targeted therapy) has greatly improved survival in COAD patients, the treatment outcomes are still unsatisfactory. Patients with similar clinical risk factors have very different prognoses and immune responses to treatment. Therefore, finding effective targets for the diagnosis and prognosis of COAD is urgently needed. IFN-γ is a pleiotropic cytokine with antiviral, antitumor, and immunomodulatory functions (15). Studies have revealed that IFN-γ acts as a proinflammatory factor to promote the growth and metastasis of COAD (16). In addition, lncRNAs have been identified to be closely associated with the development and progression of COAD. For instance, lncRNA-CYTOR promotes colon cancer epithelial-mesenchymal transition and metastasis by interacting with β-catenin (17), and lncRNA-SNHG1 promotes tumorigenicity in COAD by suppressing basal p53 levels (18). The above studies indicated that IFN-γ and lncRNAs are both involved in the progression and metastasis of COAD. However, a comprehensive systematic analysis of IFN-γ-related lncRNAs in COAD has not yet been performed. For the first time, our study analyzed the value of IFN-γ-related lncRNAs in the diagnosis and prognosis of COAD by integrated bioinformatics. Our results are expected to provide new ideas and theoretical guidance for the diagnosis and treatment of COAD.

Through mining the publicly available transcriptome sequencing data by bioinformatics analysis, many studies have constructed lncRNA signatures that predict cancer prognosis, including lung adenocarcinoma (19), gastric cancer (20), breast cancer (21), bladder urothelial carcinoma (22), hepatocellular carcinoma (23), and diffuse gliomas (24). In our study, we downloaded and integrated the gene expression data and clinical information of patients with COAD from the TCGA database, and COAD patients were divided into a training cohort and validation cohort. Through LASSO Cox regression and statistical analysis, we ultimately identified 13 IFN-γ-related lncRNAs for the construction of the prognostic signature. COAD patients were separated into high- and low-risk groups based on the median risk score. Patients in the low‐risk group had higher rates of survival than those in the high‐risk groups. Moreover, univariate and multivariate Cox regression analyses and ROC curves confirmed that the risk score based on the expression of 13 IFN-γ-related lncRNAs could predict patient prognosis independently of traditional clinical characteristics, which demonstrated the universal applicability of the risk score. The AUC values for 3-year OS in the training cohort and validation cohort were 0.792 and 0.698, respectively, indicating better predictive power compared with other similar prognostic signatures (AUC=0.585, AUC=0.63) (25, 26). In addition, the calibration curve showed that the actual observation vs. prediction rates of 1-, 3- and 5-year OS demonstrated a good consensus. Finally, we collected specimens from COAD patients who underwent surgical procedures and verified the expression of three lncRNAs (AC025165.8, RP11-324L17.1, SERTAD4-AS1) between COAD tissues and adjacent normal tissues by using qRT–PCR assays. The expression of all three lncRNAs was significantly upregulated in adjacent normal tissues in comparison to COAD tissues, which was in accordance with our bioinformatics analyses. Overall, these results implied that the IFN-γ-related lncRNA prognostic signature has better performance in predicting COAD patient prognosis and may be a potential diagnostic biomarker and therapeutic target.

LncRNA is a transcribed RNA of more than 200 nucleotides in length that is involved in a range of cell biological processes, including transcription initiation (27), transcriptional regulation (28) and chromatin modification (29). In recent years, an accumulating number of studies have indicated that lncRNAs are closely related to the initiation and progression of many kinds of cancers and have been used in the diagnosis and prognostic analysis of various cancers (30–33). Among the 13 lncRNAs in our study, FENDRR inhibits cervical cancer progression by upregulating TUBA1A in a miR-15a/b-5p-dependent manner (34). Loss and functional gain assays showed that lncRNA MYOSLID promotes gastric cancer cell proliferation and inhibits apoptosis by acting as a miR‐29c‐3p ceRNA, thereby preventing miR‐29c‐3p from binding to the target protein MCL‐1 (35). LINC00882 exerted oncogenic roles in modulating the proliferation and metastasis of hepatocellular carcinoma cells (36). In addition, we also screened 10 IFN-γ-related genes. Among the 10 IFN-γ-related genes, for instance, the increased expression of CFH interferes with proper immune surveillance and decreases the effectiveness of the immune response, thus promoting cutaneous squamous cell carcinoma progression (37). FCGR1A may be involved in the activation, regulation, or induction of immune cells and diverse physiological and pathological processes in endocervical cancer, cholangiocarcinoma, kidney renal clear cell carcinoma, and skin cutaneous melanoma (38). The FGL2-CXCL7 paracrine loop positively correlated with a higher macrophage signature and poorer prognosis in glioma patients (39). To the best of our knowledge, this study is the first to explicitly investigate the role of IFN-γ-related lncRNAs in COAD, providing a basis for further research on its molecular mechanism.

Previous studies have shown that infiltrated immune cells in the tumor microenvironment play an important role in the process of tumorigenesis and tumor progression (40, 41). In our study, we further analyzed the correlation between the IFN-γ-related lncRNA prognostic signature and the distribution of tumor-infiltrating immune cells. By applying the CIBERSORT algorithm, we characterized the abundance of 22 types of immune cells in COAD samples for a comprehensive analysis of immune cell infiltration. We found that plasma cells, activated memory CD4+ T cells, and resting memory CD4+ T cells were highly enriched in the low-risk group, whereas M0 macrophages were highly enriched in the high-risk group. Studies have shown that an increased infiltration density of CD4+ T cells in tumors is an indicator of a good prognosis (42, 43), which may explain the increased infiltration of CD4+ T cells observed in the low-risk group with better prognosis. These results suggest that IFN-γ-related lncRNAs may play a role in the immune infiltration of COAD. The clinical application of immune checkpoint inhibitors and targeted therapy for specific driver genes have dramatically improved the prognosis of patients with advanced or metastatic cancer (44, 45). In our study, there were significant differences in the expression of multiple immune checkpoint genes between the high-risk group and the low-risk group. COAD patients in the high-risk group showed higher expression of PD. L1, LAG3, TIM3 and STING, and worse prognosis than the low-risk group. These results suggest that different risk groups should be treated with different immune checkpoint blockers. In brief, this study contributes to a deeper understanding of the role of the IFN-γ-related lncRNA prognostic signature in influencing immune cell infiltration and immune checkpoint gene expression in the tumor microenvironment.

Although we combined multiple angles, datasets, and analyses to validate the robustness of our model, the research still has several limitations, and further refinement is needed. First, this study is based on a single cohort of 426 COAD patients in the publicly available TCGA database, and it is necessary to conduct more prospective clinical trials to explore its clinical value in the future. Additionally, the regulatory mechanism of the IFN-γ-related lncRNA prognostic signature in the occurrence and development of COAD is still unclear, and further research is needed. Finally, enlarging the sample size to verify our research is indispensable.

## Conclusion

In summary, the comprehensive identification and systematic analysis of IFN-γ-related lncRNAs in COAD were performed for the first time. We developed and validated a novel IFN-γ-related lncRNA signature that was closely linked to the tumour immune microenvironment and might provide potential targets for accurate prognosis of and improvement in immunotherapy for COAD patients.

## Data Availability Statement

The original contributions presented in the study are included in the article/**Supplementary Material**. Further inquiries can be directed to the corresponding author.

## Ethics Statement

The studies involving human participants were reviewed and approved by The First Affiliated Hospital of Nanchang University Ethics Committee on Medical Research. The patients/participants provided their written informed consent to participate in this study.

## Author Contributions

YX designed the study. CL performed graphing and writing. DL performed data analysis. FW and JunX performed literature search. YL, HW, and JR were responsible for language revisions. JinX, JW, and RZ helped modify articles and supervise the study. All authors reviewed the manuscript. All authors contributed to the article and approved the submitted version.

## Funding

This research was funded by the National Natural Science Foundation of China (No.81760105, No.82060108, No.81970502, No.81460115), the National Key Research and Development Program of China (2016YFC1302201), Youth Project of Jiangxi province educational department (GJJ170124), the Science and Technology Projects of Jiangxi Province (No.20161BBG70113, No.20201ZDG02007), and Leading Talent Training Plan of the Ganpo Outstanding Talents 555 Project of Jiangxi Province (2010-3-61).

## Conflict of Interest

The authors declare that the research was conducted in the absence of any commercial or financial relationships that could be construed as a potential conflict of interest.

## Publisher’s Note

All claims expressed in this article are solely those of the authors and do not necessarily represent those of their affiliated organizations, or those of the publisher, the editors and the reviewers. Any product that may be evaluated in this article, or claim that may be made by its manufacturer, is not guaranteed or endorsed by the publisher.

## Supplementary Material

Supplementary Figure 1 The correlation of the 13 IFN-γ-related lncRNAs with the clinical characteristics. **(A)** Heatmap of the associations between the expression levels of the 13 IFN-γ-related lncRNAs and clinical characteristics. **(B–D)** The relationships between the risk scores and age **(B)**, gender **(C)**, tumour stage **(D)**.

Supplementary Figure 2 Univariate Cox regression analysis **(A)** and Multivariate Cox regression analysis **(B)** of clinical characteristics and risk model scores.

Supplementary Figure 3 Kyoto Encyclopedia of Genes and Genomes (KEGG) pathway enrichment analysis of DEGs between the high- and low-risk groups.
