# Supplementary figures and images for: The Interferon Gamma-Related Long Noncoding RNA Signature Predicts Prognosis and Indicates Immune Microenvironment Infiltration in Colon Adenocarcinoma
Source: Front Oncol. 2022 Jun 7;12:876660. doi: 10.3389/fonc.2022.876660 (PMC9211770; doi:10.3389/fonc.2022.876660)

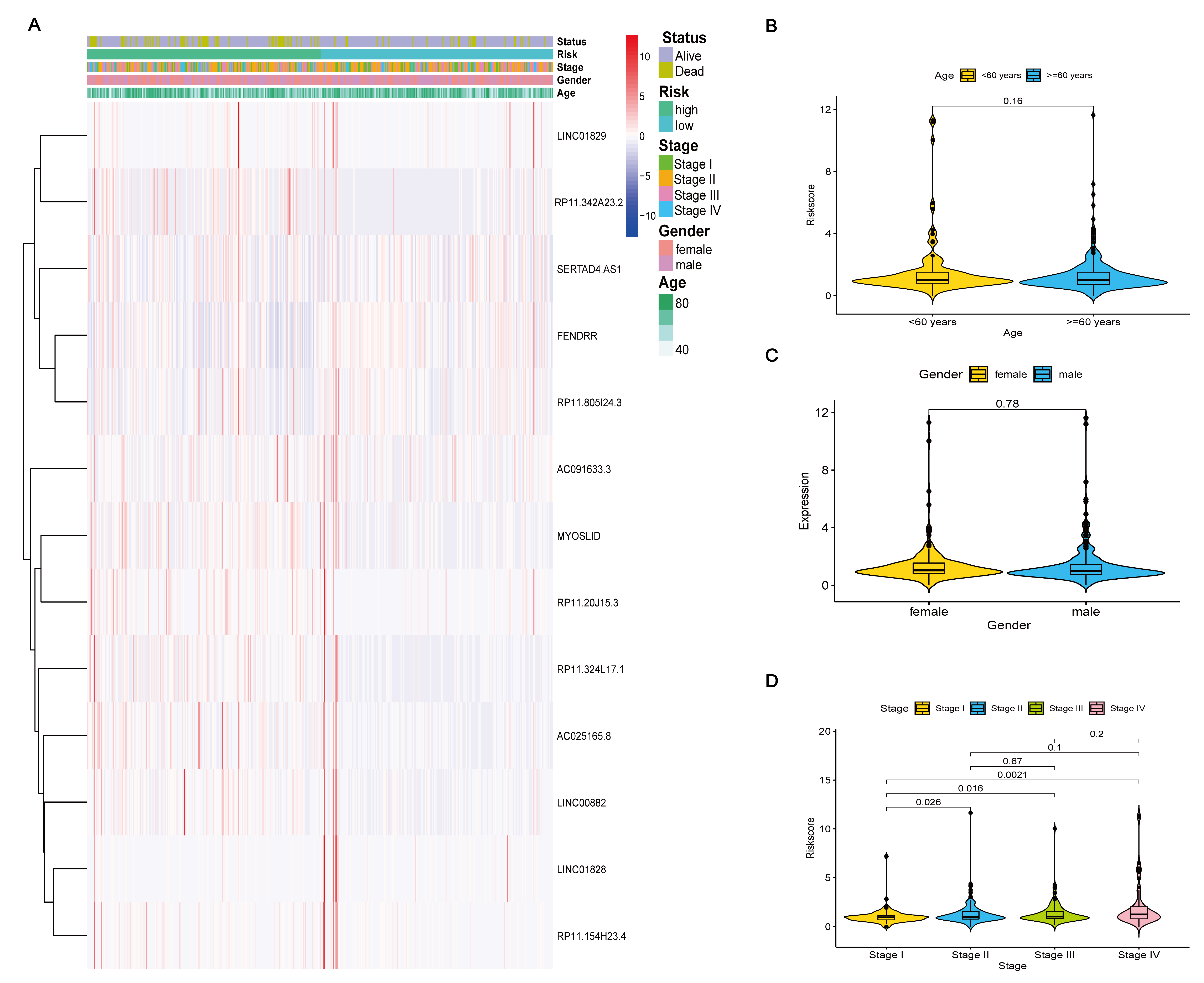

Supplement: Supplementary Figure 1 — The correlation of the 13 IFN-γ-related lncRNAs with the clinical characteristics. (A) Heatmap of the associations between the expression levels of the 13 IFN-γ-related lncRNAs and clinical characteristics. (B–D) The relationships between the risk scores and age (B), gender (C), tumour stage (D). [file Image_1.tif]

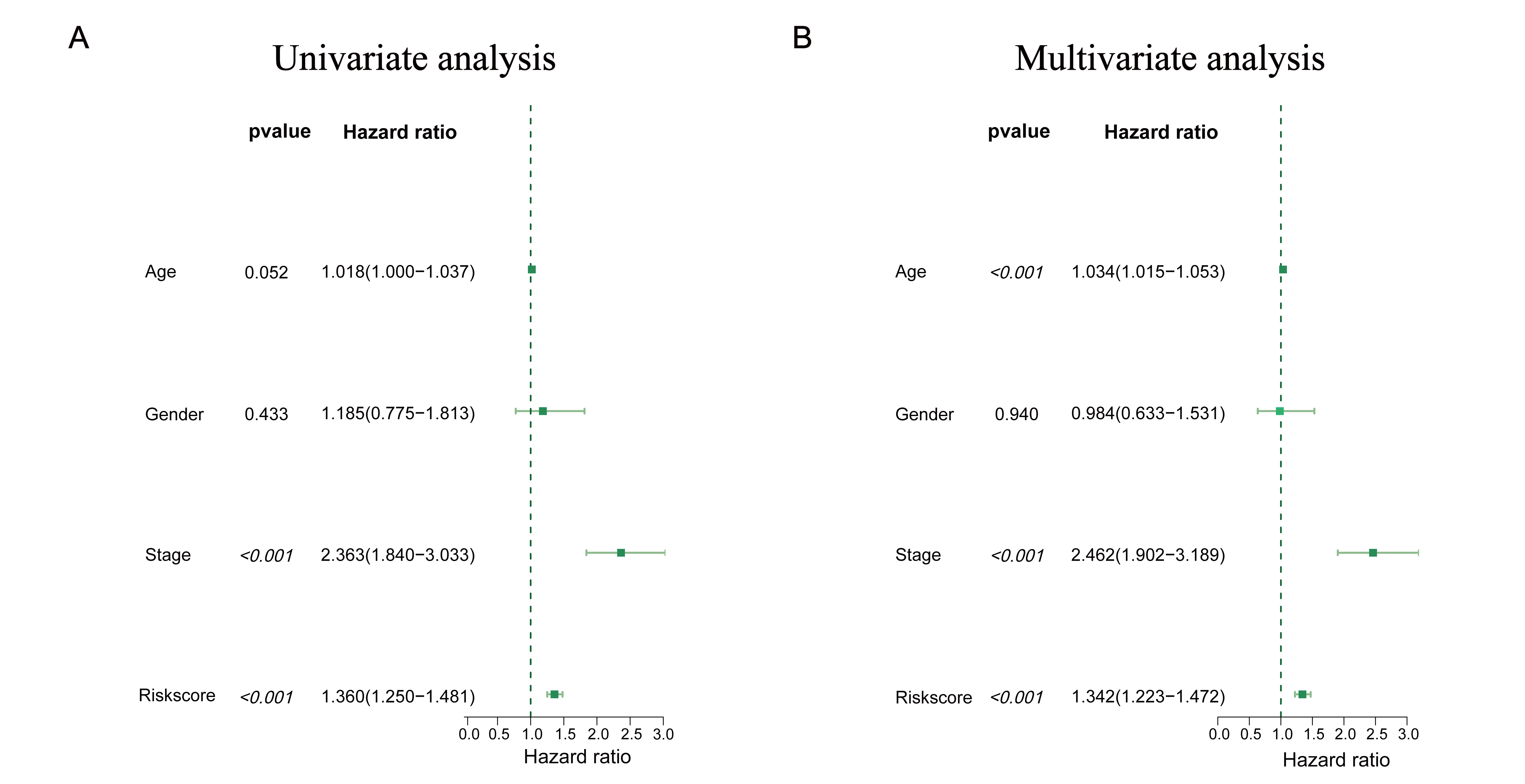

Supplement: Supplementary Figure 2 — Univariate Cox regression analysis (A) and Multivariate Cox regression analysis (B) of clinical characteristics and risk model scores. [file Image_2.tif]

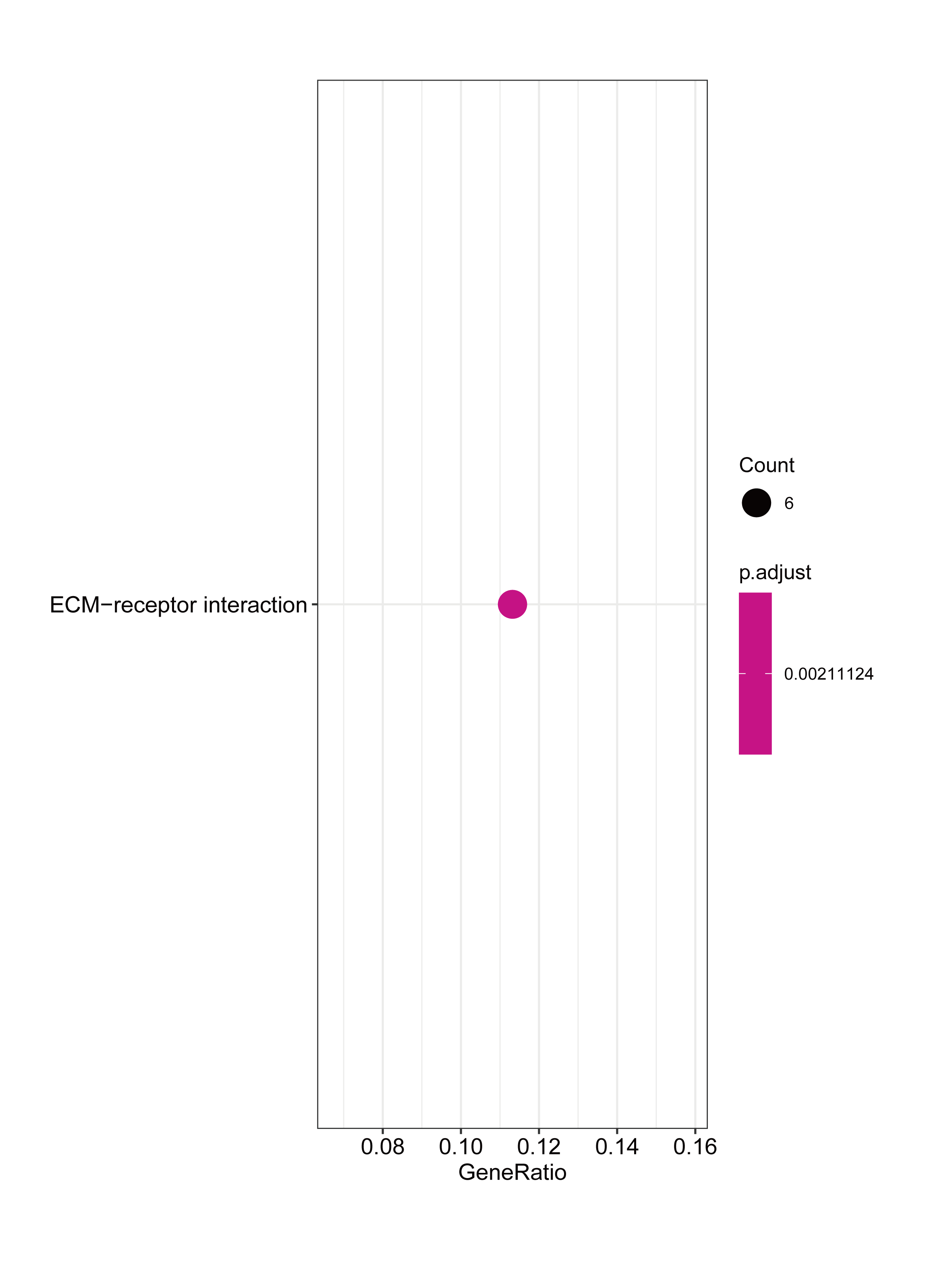

Supplement: Supplementary Figure 3 — Kyoto Encyclopedia of Genes and Genomes (KEGG) pathway enrichment analysis of DEGs between the high- and low-risk groups. [file Image_3.tif]
