# Supplementary material for: The Interferon Gamma-Related Long Noncoding RNA Signature Predicts Prognosis and Indicates Immune Microenvironment Infiltration in Colon Adenocarcinoma
Source: Front Oncol. 2022 Jun 7;12:876660. doi: 10.3389/fonc.2022.876660 (PMC9211770; doi:10.3389/fonc.2022.876660)
Supplement: Supplementary file 4 [file DataSheet_1.zip › Supplementary Tables/The download link of Supplementary Table 3.docx]

The download link of Supplementary Table 3 was

<https://www.jianguoyun.com/p/DSOAZhkQy4H4CRi97ZUE>
